# Effect of sarcopenia in predicting postoperative mortality in emergency laparotomy: a systematic review and meta-analysis

**DOI:** 10.1186/s13017-022-00440-0

**Published:** 2022-06-25

**Authors:** Tao-ran Yang, Kai Luo, Xiao Deng, Le Xu, Ru-rong Wang, Peng Ji

**Affiliations:** 1 Department of Anesthesiology, West China Hospital, Sichuan University, No. 37, Guoxue Xiang, Chengdu, 610041 Sichuan China; 2 Department of Anesthesiology, West China Second University Hospital, Sichuan University, Chengdu, Sichuan China; 3 Key Laboratory of Birth Defects and Related Diseases of Women and Children, Ministry of Education, Sichuan University, Chengdu, Sichuan China; 4 Department of Critical Care Medicine, West China Hospital, Sichuan University, No. 37, Guoxue Xiang, Chengdu, 610041 Sichuan China

**Keywords:** Sarcopenia, Emergency laparotomy, Postoperative mortality

## Abstract

**Background:**

While emergency laparotomy has been associated with high rates of postoperative mortality and adverse events, preoperative systematic evaluation of patients may improve perioperative outcomes. However, due to the critical condition of the patient and the limited operation time, it is challenging to conduct a comprehensive evaluation. In recent years, sarcopenia is considered a health problem associated with an increased incidence of poor prognosis. This study aimed to investigate the effect of sarcopenia on 30-day mortality and postoperative adverse events in patients undergoing emergency laparotomy.

**Methods:**

We systematically searched databases including PubMed, Embase, and Cochrane for all studies comparing emergency laparotomy in patients with and without sarcopenia up to March 1, 2022. The primary outcome was of 30-day postoperative mortality. Secondary outcomes were the length of hospital stay, the incidence of adverse events, number of postoperative intensive care unit (ICU) admissions, and ICU length of stay. Study and outcome-specific risk of bias were assessed using the Quality in Prognosis Studies (QUIPS) tool. We rated the certainty of evidence using the Grading of Recommendations, Assessment, Development and Evaluations (GRADE).

**Result:**

A total of 11 eligible studies were included in this study. The results showed that patients with sarcopenia had a higher risk of death 30 days after surgery (OR = 2.42, 95% CI = 1.93–3.05, *P* < 0.00001). More patients were admitted to ICU after surgery (OR = 1.58, 95% CI = 1.11–2.25, *P* = 0.01). Both the ICU length of stay (MD = 0.55, 95% CI = 0.05–1.06, *P* = 0.03) and hospital length of stay (MD = 2.33, 95% CI = 1.33–3.32, *P* < 0.00001) were longer in the sarcopenia group. The incidence of postoperative complications was also significantly higher in patients with sarcopenia (OR = 1.78, 95% CI = 1.41–2.26, *P* < 0.00001).

**Conclusion:**

In emergency laparotomy, sarcopenia was associated with increased 30-day postoperative mortality. Both the lengths of stay in the ICU and the total length of hospital stay were significantly higher than those in non-sarcopenic patients. Therefore, we concluded that sarcopenia can be used as a tool to identify preoperative high-risk patients, which can be considered to develop new postoperative risk prediction models.

*Registration number* Registered on Prospero with the registration number of CRD42022300132.

**Supplementary Information:**

The online version contains supplementary material available at 10.1186/s13017-022-00440-0.

## Introduction

Emergency laparotomy is a time-sensitive procedure with a high mortality range from 8.8 to 18.6% [1–3], which is much higher than elective surgeries [4–6]. Elderly patients suffering worse morbidity and mortality following emergency laparotomy have been contributed to multiple medical comorbidities and nutrition status [7]. Effective identification of high-risk patients has been the key to improving perioperative outcomes [5]. However, due to the complexity of the patient's condition, the different variety of surgical types, and the tight operation preparation time, it has been challenging to predict the outcome after emergency surgery based on preoperative information. Several previous studies have used various scoring systems to predict the risk of emergency laparotomy [8, 9]. However, when patients’ condition is too critical to complete functional tests and answer related questions in emergency conditions, the scores are usually subjective and inaccurate [10, 11]. Therefore, it is an urgent requirement to develop a new assessment tool to identify the patient at risk of emergency laparotomy and guide optimal perioperative management [9]. Body composition also plays an important role in predicting treatment outcomes in patients following surgery. Sarcopenia has been observed to be a strong prognostic indicator for perioperative complications [12, 13], including cognitive impairment [14], fractures [15], mental disorders [16], and even survival [17, 18].

Sarcopenia refers to the progressive and global decline in skeletal muscle mass and strength associated with aging, immobility, or illness status [19]. Although there are discrepancies in the diagnostic criteria of sarcopenia in different countries and regions, the cross-sectional area of the lumbar muscle on an abdominal computed tomography (CT) scan is an internationally recognized simple and reliable indicator [20]. It is assessed by measuring muscle mass at the level of the L3 vertebra, which has made a preoperative assessment of psoas major area (PMA) and total skeletal muscle area (SMA) possible based on the routine examination of an abdominal CT scan [21].

Studies have shown that sarcopenia increases the incidence of adverse events and mortality after elective esophageal cancer surgery, gastrectomy, and pancreatic surgery [22–24]. However, there has been no definite conclusion on the impact of 30-day mortality and postoperative adverse events on emergency laparotomy. Hajibandeh et al. pointed out that sarcopenia can be used to predict mortality in both emergency and elective abdominal surgeries [25]. However, only four studies of emergency surgery were included, so it is unconvincing to draw reliable conclusions. We, therefore, performed further analysis to assess the impact of sarcopenia on 30-day mortality and postoperative complications in patients following emergency laparotomy.

## Methods

This systematic review and meta-analysis were prepared in accordance with the latest PRISMA requirements and was registered with Prospero (registration number: CRD42022300132) [26]. Two researchers (T.Y. and K.L.) searched databases such as PubMed, Embase, and Cochrane. The search date was as of March 1, 2022. The search was not limited to language and region, and we provided a PRISMA checklist. PubMed's search strategy can be found in Table 1.

**Table 1:** Search strategy of PubMed

Search	Query
#1	"Surgical Procedures, Operative"[MeSH Terms]
#2	"Operative Procedure"[All Fields] OR "Procedure, Operative"[All Fields] OR "Surgical Procedure, Operative"[All Fields] OR "Operative Surgical Procedures"[All Fields] OR "Procedure, Operative Surgical"[All Fields] OR "Surgical Procedures"[All Fields] OR "Procedure, Surgical"[All Fields] OR "Surgical Procedure"[All Fields] OR "Operative Surgical Procedure"[All Fields] OR "Surgery, Ghost"[All Fields]
#3	#1 OR #2
#4	"Abdomen"[MeSH Terms] OR "Abdomens"[All Fields]
#5	#3 AND #4
#6	"Laparotomy"[MeSH Terms] OR "Laparotomies"[All Fields] OR "Minilaparotomy"[All Fields] OR "Minilaparotomies"[All Fields]
#7	#5 OR #6
#8	"Emergencies"[MeSH Terms] OR "Emergency"[All Fields]
#9	#7 AND #8
#10	"Sarcopenia"[MeSH Terms] OR "Sarcopenias"[All Fields]
#11	#9 AND #10

### Study selection

We aimed to include all the studies comparing patients with sarcopenia and non-sarcopenic patients following emergency laparotomy. Inclusion and exclusion criteria were conducted in advance. The inclusion criteria were as follows:

1. Age ≥ 18 years old;
2. Patients were treated by emergency laparotomies;
3. Preoperative abdominal/pelvic CT data were present.

Emergency operations in this study included segmental or total colectomy, small bowel resection, open cholecystectomy, open appendectomy, abscess drainage, exploratory laparotomy, etc. Our exclusion criteria were:

1. Elective surgery;
2. Traumatic abdominal surgery;
3. Emergency abdominal vascular surgery;
4. Studies with an unclear diagnosis of sarcopenia.

Two of our investigators (T.Y. and K.L.) selected studies that were compliant with a full-text reading by reviewing titles and abstracts. Any disagreements between investigators were independently resolved (R.W.). In addition, we searched the World Health Organization International Clinical Trials Registry and queried bibliographic lists of relevant articles and reviews for further potentially eligible studies.

### Data extraction

Two investigators (T.Y. and X.D.) independently extracted the following data: author name and publication year, literature type, the sample size for exposure and control groups, diagnostic criteria for sarcopenia, and characteristics of the included population. During this process, all disagreements were resolved by discussion, and if necessary, a third author (P.J.) was consulted.

We extracted data directly from the original text for synthesis. If the data were presented in the form of a graph and could not be directly extracted, we used a Plot digitizer or contacted the corresponding author. We extracted continuous results as the mean and standard deviation, and if the median was displayed, we converted the median and interquartile range to mean and standard deviation using the statistical formula [27, 28].

### Quality assessment and risk of bias

Two investigators (T.Y. and K.L.) independently used the Quality in Prognosis Studies (QUIPS) critical assessment tool to assess the risk of bias for including studies [29, 30]. This tool is designed for systematic reviews of prognostic factor studies. The scale mainly includes study participation, study attrition, prognostic factor measurement, outcome measurement, study confounding, and statistical analysis and reporting. Each domain is assessed against criteria, thereby resulting in a rating of ‘high,’ ‘moderate,’ or ‘low’ risk of bias. Any discrepancies between investigators were discussed at a consensus meeting, and any further disagreement was resolved by discussion with a third investigator.

We used the GRADE (Grading of Recommendations, Assessment, Development, and Evaluation) [31] approach to assess the quality of evidence for 30-day postoperative mortality, complication rates, ICU admission, the length of ICU stay, and the length of hospital stay. We rated the quality of the evidence as 'high', 'moderate', 'low', and 'very low' based on risk of bias, inconsistency, indirectness, imprecision, and other considerations. And we used GRADEpro to generate the Summary of Finding (SoF).

### Outcome

The primary outcome was 30-day mortality after emergency laparotomy. Secondary outcomes included incidence of postoperative complications, number of postoperative ICU admissions, and ICU and hospital length of stay, respectively.

### Data analysis

We used Revman 5.3 for meta-analysis. For continuous variables, we used mean difference (MD) and 95% confidence interval (CI). For binary variables, the odds ratio (OR) value for statistics was implemented. In this study, considering that there was always heterogeneity in terms of the type of surgery, surgical technique, and experience of surgeons, all results in this study were performed using a random-effects model. For assessing the outcome, we performed a sensitivity analysis by using the leave-one-out approach to identify the possible sources of heterogeneity.

## Result

**Fig 1.**
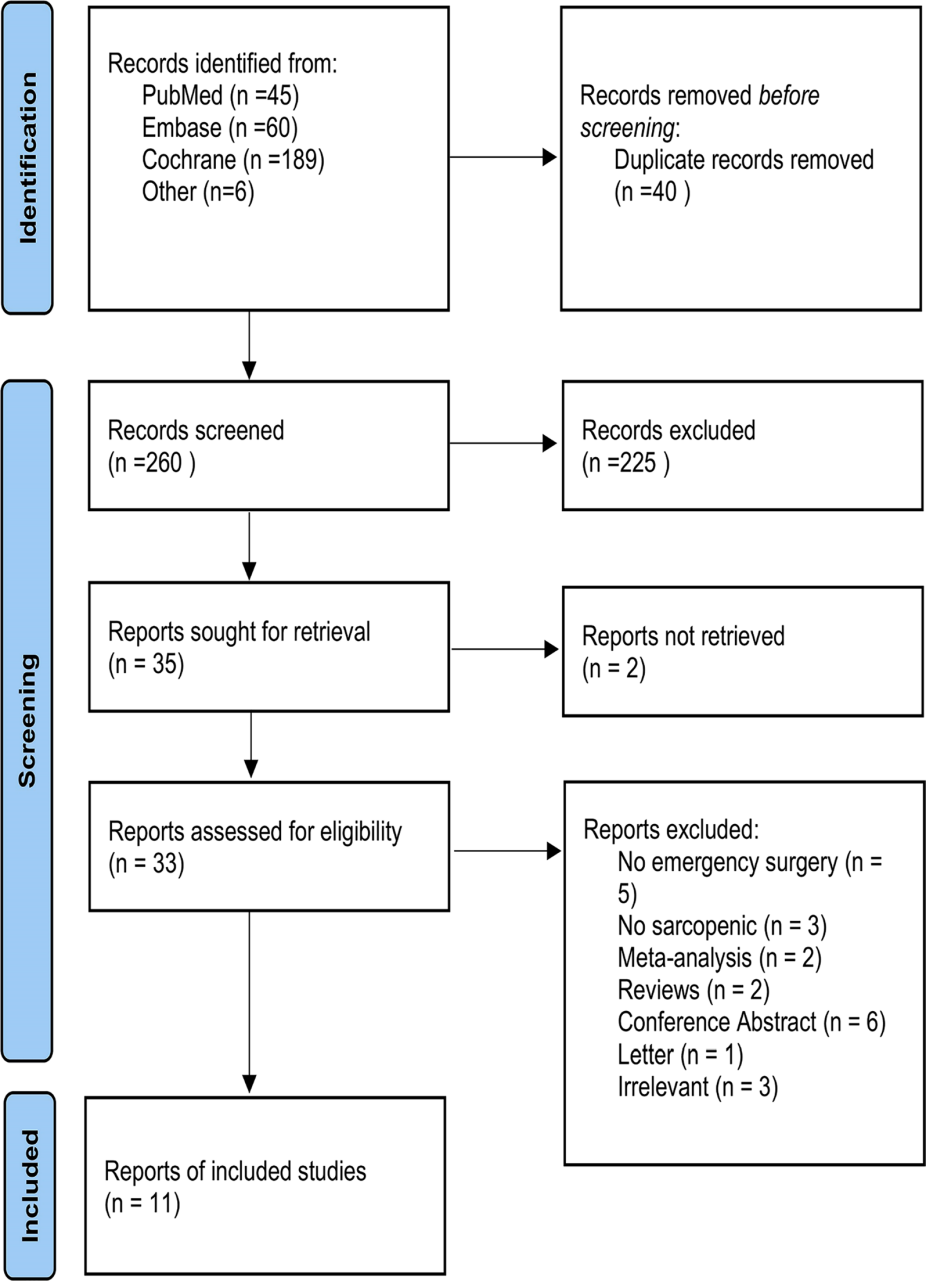
Flowchart showing selection of articles for review

Through systematic database searching, we identified 300 articles. After screening to remove duplicate literature, there were 260 articles in total. Thirty-three articles were reviewed for full text following evaluation of the titles and abstracts. Twenty-two articles were excluded due to non-emergency surgery and unclear diagnosis. Review and conference abstract were also excluded in terms of the study design. Overall, 11 articles with 3795 patients were included for further analysis. Fig 1 depicts a flowchart of the study selection process.

Table 2 summarizes the characteristics of the 11 articles that met the inclusion criteria. The total sample size ranged from 80 to 967, all of which were retrospective studies [5, 17, 18, 32–39]. Of the studies, 10 of them examined muscle mass at the level of the L3 vertebral body on CT scans to diagnose sarcopenia [5, 17, 32–37, 39] and only one assessed the muscle mass at the level of the L4 vertebral body on CT scans [38].

**Table 2:** Characteristics of included studies

Author, year	Study design	Total Sample	Diagnose	Age	BMI	Sample	The included surgery	Diagnostic criteria	
Mohammad 2018	Retrospective study	*n* = 452	Sarcopenia	62 (11.3)	23 [22–28]	*n* = 113	Emergency laparotomy surgery	Using CT to assess the psoas muscle at the L3 level	
			No sarcopenia	55 (9.1)	25 [23–29]	*n* = 339			
Colin 2021	Retrospective study	*n* = 80	Sarcopenia	65.7(median 67.5)	UK	*n* = 20	Emergency laparotomy surgery	Using CT to assess the psoas muscle at the L3 level	
			No sarcopenia	59.6(median 60.0)	UK	*n* = 60			
Trotter 2018	Retrospective study	*n* = 248	Sarcopenia	72 (15.7)	24.4 (6.1)	*n* = 61	Emergency laparotomy surgery	Using CT to assess the psoas muscle at the L3 level	
			No sarcopenia	70 (16.0)	25.6 (6.6)	*n* = 187			
Matsushima 2017	Retrospective study	*n* = 89	Sarcopenia	54 [50–62]	UK	*n* = 32	Acute colonic diverticulitis	Using CT to assess the psoas muscle at the L3 level	
			No sarcopenia	44 [36–52]	UK	*n* = 57			
Samer 2019	Retrospective study	*n* = 283	Sarcopenia	78.92(7.66)	UK	*n* = 73	Emergency laparotomy surgery	Using CT to assess the psoas muscle at the L3 level	
			No sarcopenia	77.56(7.75)	UK	*n* = 210			
Rebecca 2016	Retrospective study	*n* = 593	Sarcopenia	65.65(15.68)	23.69(5.53)	*n* = 197	Emergency laparotomy surgery	Using CT to assess the psoas muscle at the L4 level	
			No sarcopenia	58.14(16.22)	29.67(8.01)	*n* = 396			
Du 2014	Retrospective study	*n* = 100	Sarcopenia	84.3(3.9)	24 [22–27]	*n* = 73	Emergency general surgical operation	Using CT to assess the psoas muscle at the L3 level	
			No sarcopenia	83.6(2.9)	25 [24–28]	*n* = 27			
Brandt 2019	Retrospective study	*n* = 150	Sarcopenia	UK	UK	*n* = 38	Emergency laparotomy surgery	Using CT to assess the psoas muscle at the L3 level	
			No sarcopenia	UK	UK	*n* = 112			
Lisa 2018	Retrospective study	*n* = 967	Sarcopenia	70.3(14.7)	25.0(5.6)	*n* = 241	Acute care surgery	Using CT to assess the psoas muscle at the L3 level	
			No sarcopenia	61.2(16.8)	30.7(8.7)	*n* = 726			
Samantha 2021	Retrospective study	*n* = 536	Sarcopenia	75 [68–81]	23.4 [20.2–27.1]	*n* = 241	Emergency laparotomy surgery	Using CT to assess the psoas muscle at the L3 level	
			No sarcopenia	68 [54–77]	26.2 [22.7–30.1]	*n* = 726			
Rangel 2016	Retrospective study	*n* = 297	Sarcopenia	78 [74–84]	22 [20–27]	*n* = 75	Acute abdominal surgery	Using CT to assess the psoas muscle at the L3 level	
			No sarcopenia	78 [74–83]	27 [24–31]	*n* = 222			

The number represents the mean (standard deviation) or median (interquartile range) or mean (median)

UK, unknown; BMI, body mass index

### Risk of bias

Table 3 shows the risk of bias for the 11 included studies assessed according to the QUIPS tool, of which almost all were rated moderate to high for potential risk of bias in study attrition and study confounding domains. This was the most common methodological weakness.

**Table 3:** Risk of bias summary: judgment of each domain for all included studies using the Quality of Prognostic Studies (QUIPS) tool

Study	Study participation	Study attrition	Prognostic factor measurement	Outcome measurement	Study confounding	Statistical analysis and reporting
Mohammad 2018	Moderate	Moderate	Low	Low	Moderate	Moderate
Colin 2021	Low	High	Moderate	Low	High	Low
Trotter 2018	Low	Moderate	Low	Low	High	Low
Matsushima 2017	Low	High	Low	Low	Moderate	Low
Samer 2019	Low	Moderate	Low	Low	High	Moderate
Rebecca 2016	Low	High	Low	Low	High	Low
Du 2014	Low	Moderate	Low	Low	High	Moderate
Brandt 2019	Low	High	Low	Low	High	Low
Lisa 2018	Low	High	Low	Low	Moderate	Low
Samantha 2021	Low	High	Low	Low	Moderate	Low
Rangel 2016	Low	Moderate	Low	Low	Moderate	High

### Quality of evidence

**Fig 2.**
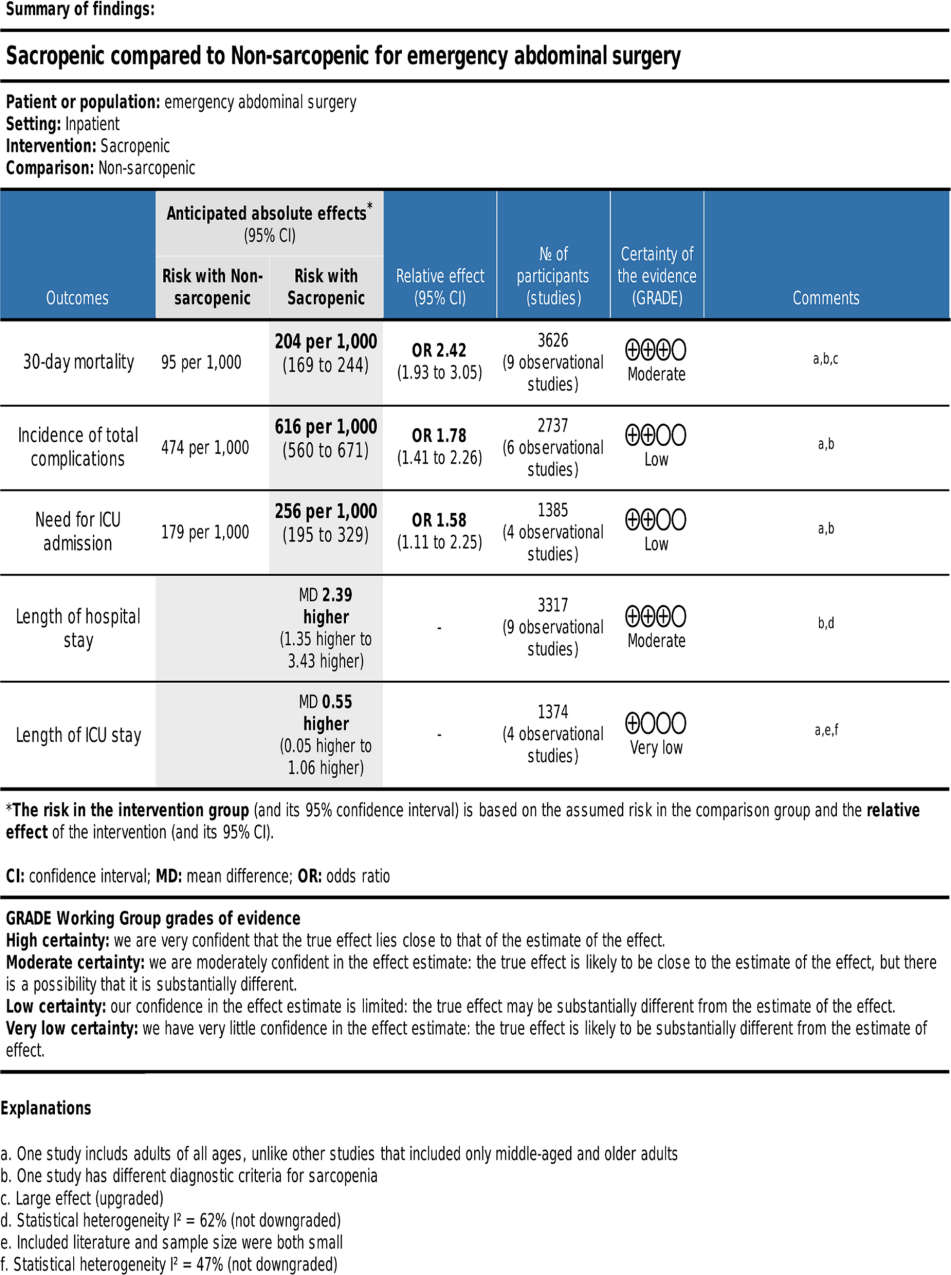
Certainty of the evidence and summary of findings

Based on the GRADE approach, we found the moderate quality of evidence for 30-day postoperative mortality and length of hospital stay. The quality of evidence was low for the need for ICU admission and the incidence of postoperative complications, and the quality of evidence for the length of stay in the ICU was very low (Fig. 2).

### Primary outcome

#### 30-day mortality

**Fig 3.**
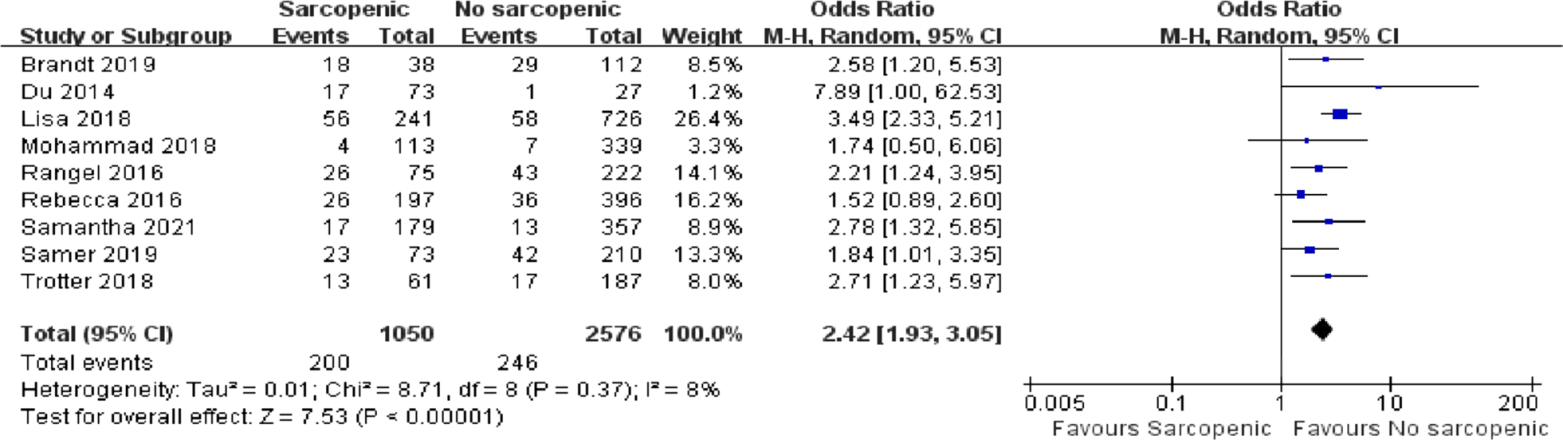
Forest plot showing 30-day mortality

Nine articles with a total of 3626 patients reported 30-day mortality following emergency laparotomy [5, 17, 32–34, 36–39]. Compared with non-sarcopenic patients, sarcopenic patients had a higher risk of death 30 days after surgery (Fig. 3; OR = 2.42, 95% CI = 1.93–3.05, *P* < 0.00001). There was slight heterogeneity among the included kinds of literature (*I*^2^ = 8%, *P* = 0.37).

### Secondary outcome

#### Length of ICU stay

**Fig 4.**
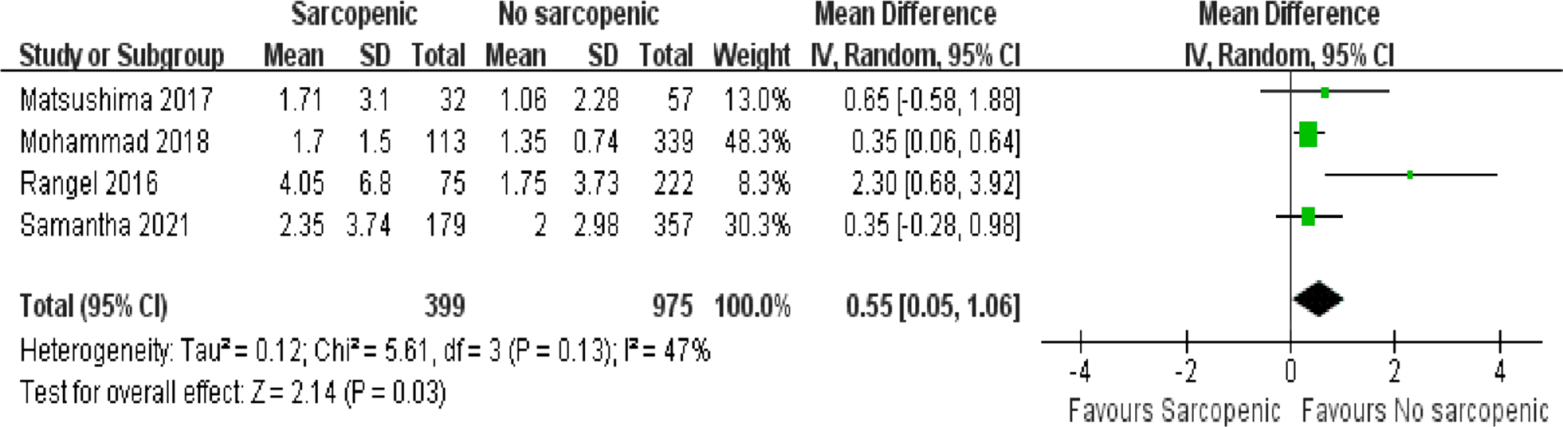
Forest plot showing the length of ICU stay

There were four articles documenting the length of stay in the ICU, in which data were reported for 1374 cases [5, 34–36]. The sarcopenia group was found to have longer ICU stays (Fig. 4; MD = 0.55, 95% CI = 0.05–1.06, *P* = 0.03), with acceptable heterogeneity between articles (*I*^2^ = 47%, *P* = 0.13).

### Need for ICU admission

**Fig 5.**
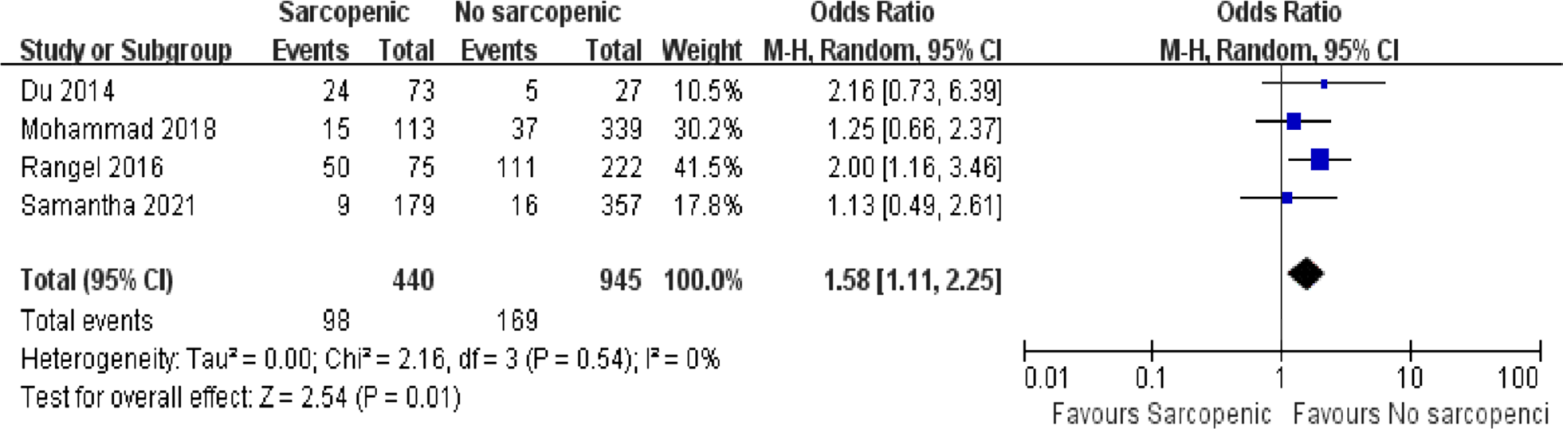
Forest plot showing the need for ICU admission

Four articles with a total of 1385 patients documented the number of postoperative ICU admissions required [5, 34, 36, 37]. There was a significant difference between sarcopenia and non-sarcopenic groups (Fig. 5; OR = 1.58, 95% CI = 1.11–2.25, *P* = 0.01). Patients diagnosed with sarcopenia were more likely to be admitted to the ICU after emergency surgery. Among all included articles, the identity was high and there was no heterogeneity (*I*^2^ = 0%, *P* = 0.54).

### Total complications

**Fig 6.**
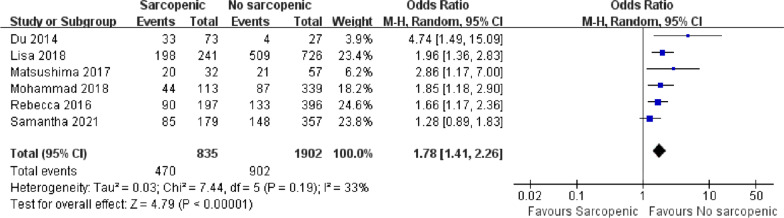
Forest plot showing the incidence of total complications

A total of six articles reported the incidence of postoperative complications in the emergency department, with a total of 2737 patients [5, 17, 35–38]. There was low heterogeneity among the included articles (*I*^2^ = 33%, *P* = 0.19). Patients with sarcopenia were more likely to have certain complications after emergency laparotomy (Fig. 6; OR = 1.78, 95% CI = 1.41–2.26, *P* < 0.00001).

### Length of hospital stay

**Fig 7.**
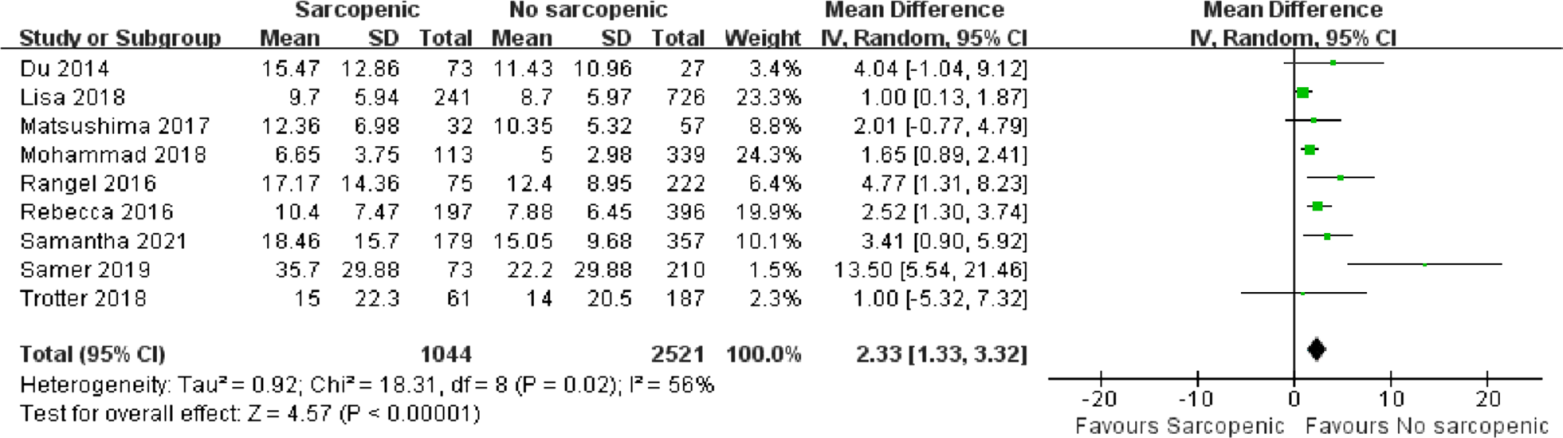
Forest plot showing the length of hospital stay

There were nine articles with a total of 3565 patients reporting postoperative hospital stays [5, 17, 32–38]. There was a significant difference in the length of hospital stay between the sarcopenia group and the non-sarcopenic group (Fig. 7; MD = 2.33, 95% CI = 1.33–3.32, *P* < 0.00001; *I*^2^ = 56%). This suggested that patients with sarcopenia had longer hospital stays after emergency surgery. Due to significant heterogeneity, we further performed a sensitivity analysis. In the study by Samer, only the duration of hospital stay in patients who survived within the first 30 days after surgery was counted [32]. After excluding this article, there was still a significant difference in the length of hospital stay between the two groups, but the heterogeneity between articles was reduced (Table 4; MD = 1.94, 95% CI = 1.23–2.65, *P* < 0.00001; *I*^2^ = 30%).

**Table 4:** The sensitivity analysis of the length of hospital stay

Study	Statistics with study removed					
	MD	Lower limit	Upper limit	*Z* value	*P* value	*I*^2^ value
Du 2014	2.28	1.25	3.3	4.34	0.01	60%
Lisa 2018	2.77	1.58	3.96	4.56	0.06	49%
Matsushima 2017	2.41	1.31	3.5	4.29	0.01	62%
Mohammad 2018	2.78	1.36	4.2	3.84	0.01	61%
Rangel 2016	2.12	1.14	3.1	4.25	0.03	54%
Rebecca 2016	2.37	1.17	3.57	3.86	0.02	58%
Samantha 2021	2.2	1.15	3.26	4.1	0.02	58%
Samer 2019	1.94	1.23	2.65	5.35	0.19	30%
Trotter 2018	2.39	1.35	3.43	4.49	0.01	62%

MD, mean difference

## Discussion

Emergency laparotomy surgery had high morbidity and mortality. There were few studies investigating the impact of sarcopenia in patients following emergency laparotomy. Our study aimed to assess the risk of death after emergency laparotomy in patients with preoperative sarcopenia. We performed a systematic review and meta-analysis. Eleven eligible studies involving 3795 patients were included. The results showed that sarcopenia was associated with increased postoperative 30-day mortality. There was mild heterogeneity among the nine studies, which were considered high quality. In addition, patients with sarcopenia significantly increased postoperative ICU duration, number of ICU admissions, incidence of postoperative complications.

Patients with preoperative sarcopenia had significantly longer hospital length of stay, but there was large heterogeneity between studies. One of the studies included patients who survived more than or equal to 30 days after surgery in the overall length of stay calculation. The author believed that the impact of early death should be excluded. However, patients that died during the study period were included so as to not overestimate the total duration result. In fact, hospital length of stay in this study was significantly longer than others, which showed that overstated time was a variable of heterogeneity in our study. Considering ICU admissions and hospital length of stay, it was reasonable to presume that patients with sarcopenia following emergency laparotomy were at risk of high medical costs and excessive heavy illness burden.

Our systematic review and meta-analysis independently investigated the association of sarcopenia with prognosis after emergency laparotomy. An efficient and simple assessment tool to identify high-risk surgical patients may be extremely valuable for medical teams’ awareness, especially in time-sensitive situations. Our findings were consistent with previous observative studies confirming that sarcopenia was associated with increased 30-day postoperative mortality in patients undergoing emergency laparotomy [25, 40, 41]. Some studies identified the effect of sarcopenia on mortality and morbidity after elective and emergency abdominal surgeries. However, the quality of evidence is low due to the small number of included studies and participants [25]. In recent years, the impact of sarcopenia on postoperative outcomes has received extensive attention, especially in emergency laparotomy. Therefore, we performed an update of this topic including 11 studies of 3795 patients, and we used the QUIPS and GRADE tools, respectively, to assess the risk of bias and quality of evidence in the included studies. In our study, heterogeneity was low with a dramatically expanded sample size, and the methodological quality of the studies in our review was reliable, increasing the representativeness, and generalizability of our conclusions.

Previous studies found that sarcopenia was associated with multiple adverse outcomes, including falls, functional decline, and postoperative mortality [42]. Sarcopenia was assessed by abdominal computed tomography (CT) L3 pyramid or L4 pyramid total psoas area (TPA) or total psoas index (TPI). TPA < 3.64 cm/m^2^ in women and TPA < 4.55 cm/m^2^ in men or TPI < 1.50 cm/m^2^ in women and TPI < 2.16 cm/m^2^ in men were defined as sarcopenia [43–46]. Therefore, sarcopenia by abdomen CT scan was used to easily identify the high-risk patients without evaluating them from complicated scales or questionnaires, which is both costly for time and inaccurate in terms of patients’ severeness and degree of cooperation. Meanwhile, most of the patients treated by emergency laparotomy had abdominal CT scan before surgeries. Therefore, sarcopenia was simple, objective, and efficient to be a perioperative risk stratification tool in emergency clinical practice.

The reasons for poor prognosis of patients with sarcopenia after emergency laparotomy were multifactorial, including preoperative frailty, previous malnutrition, and complex comorbidities. In addition, emergency laparotomy surgeries were often insufficiently prepared due to rapid and even life-threatening disease progression. In the postoperative stage, surgical strikes, pain, respiratory failure, circulatory compromise, sepsis, and multiple organ dysfunction also increased the incidence of postoperative complications and mortality.

Although CT-identified sarcopenia can help to predict perioperative risk, we cannot improve patient outcomes through preoperative nutrition and physical activity, as practiced in elective surgery [47]. To these patient groups, we likely pay more attention throughout treatment, such as complications prevention, nutrition intervention in early post-procedure phase, and even immediate intensive care post-surgery. Therefore, preoperative diagnosis of sarcopenia undoubtedly provided important predictive information for general medical management strategies, nursing goals, family awareness, and rehabilitation expectations [48].

There were some limitations in our study. Firstly, the 11 included studies were all retrospective cohort studies. The retrospective design caused them to solely assess muscle mass without measurement of muscle function (grip strength, walking speed, physical activity), which may exaggerate the predictive power of sarcopenia. In our opinion, given that sarcopenia is a serious disease state, we would rather overestimate than underdiagnose. Secondly, there were no clear definition and classification of surgical types in the included literature, so we could not perform subgroup analysis according to surgical procedures specifically, which may have influenced the results of this study. Further research is required to be addressed on specific procedures later. In addition, positive results were more likely to be published and there may be a risk of reporting bias. Most important, in the emergency setting, we have demonstrated that sarcopenia can effectively predict adverse postoperative outcomes.

Recently, some studies argued that patients with sarcopenia cannot be diagnosed by preoperative CT efficiently because of the costs, radiations, and body position restrictions. The point-of-care ultrasound had the advantages of high operability, repeatability, and convenient portability (49). Therefore, point-of-care ultrasound instead of CT scan may be a future research direction to assess the feasibility of sarcopenia diagnosis before emergency surgeries. Exploration of randomized controlled trials is also needed if early targeted interventions on sarcopenia can improve patient outcomes based on our prediction findings.

## Conclusion

We found that preoperative CT scan-derived sarcopenia was associated with increased postoperative 30-day mortality and ICU admissions. ICU and hospital length of stay and incidence of postoperative adverse events were also significantly elevated. Further research should demonstrate if dedicated intervention for sarcopenia will improve patient outcomes.

## Supplementary Information

**Additional file 1.** PRISMA checklist.

## Data Availability

The datasets generated and analyzed during the current study are available from the corresponding author on reasonable request.

## Abbreviations

OR: Odds ratio
CI: Confidence interval
MD: Mean difference
PRISMA: Preferred reporting items for systematic reviews and meta-analyses
MeSH: Medical Subject Headings
ICU: Intensive care unit
PMA: Psoas major area
SMA: Skeletal muscle area
CT: Computed tomography
TPA: Total psoas area
TPI: Total psoas index
QUIPS: Quality in prognosis studies
GRADE: Grading of Recommendations, Assessment, Development and Evaluations
SoF: Summary of finding
